# Using Plasma Amyloid Beta Oligomer to Screen in Alzheimer’s Disease: A Pilot Study

**DOI:** 10.3390/ijms27020846

**Published:** 2026-01-14

**Authors:** Pin-Chieh Hsu, Jia-Ying Yang, Ling-Chun Huang, Yuan-Han Yang

**Affiliations:** 1 School of Post-Baccalaureate Medicine, College of Medicine, Kaohsiung Medical University, Kaohsiung 80756, Taiwan; a120148118@gmail.com (P.-C.H.); yangwish0630@gmail.com (J.-Y.Y.); hlg711022@gmail.com (L.-C.H.); 2 Department of Neurology, Kaohsiung Medical University Gangshan Hospital, Kaohsiung Medical University, Kaohsiung 80756, Taiwan; 3 Department of Neurology, Kaohsiung Medical University Hospital, Kaohsiung Medical University, Kaohsiung 80756, Taiwan; 4 Neuroscience Research Center, Kaohsiung Medical University, Kaohsiung 80756, Taiwan

**Keywords:** Alzheimer’s disease, amyloid-beta oligomer, early detection, blood-based diagnosis, plasma biomarkers

## Abstract

Previous studies have shown that plasma amyloid-beta oligomers (AβOs), the toxic form of amyloid-beta (Aβ), are a critical issue in the development or worsening of Alzheimer’s disease (AD) and can be regarded as a blood marker for screening in dementia. We examined plasma AβOs with their related biomarkers in a case–control study to clarify these issues. A total of 16 patients diagnosed with Alzheimer’s dementia (AD) and 16 cognitively normal controls (NCs) were recruited to compare their plasma biomarkers, AβO, Aβ_1-40_, and Aβ_1-42_, also referring to other parameters like APOE ε4 status, Clinical Dementia Rating^®^-Sum of Boxes (CDR^®^-SB), and Mini Mental Status Examination (MMSE) scores. In plasma concentrations of Aβ_1-40_, Aβ_1-42_, and AβO, the mean concentrations were significantly different between the two groups. There is a significant increase in the concentrations of Aβ_1-40_ and AβO, while Aβ_1-42_ is decreased in individuals with AD compared to NC. AβO was statistically associated with the Aβ_1-40_ and Aβ_1-42_/Aβ_1-40_ ratio. Higher plasma concentrations of AβO were significantly associated with AD compared to non-dementia controls. This suggests that AβOs can be potential plasma biomarkers to screen in AD. However, a study recruiting more individuals is necessary to examine the association, if any.

## 1. Introduction

Alzheimer’s disease (AD) is a neurodegenerative disease, characterized by the buildup of pathological amyloid-beta (Aβ) aggregates within the brain [1]. The production and deposition of the Aβ peptide are widely believed to drive the pathogenesis of AD [2]. According to the National Institute on Aging–Alzheimer’s Association (NIA-AA) Research Framework [3], changes in Aβ can be detected during the preclinical stage, when individuals are still cognitively normal. Because brain damage that occurs later in the disease is often irreversible, treatments aimed at slowing or stopping the disease may be most effective if started early. For this reason, improving the ability to screen people with early-stage AD is important.

Currently, diagnosis and management of early AD are largely guided by clinical symptoms [4]. Cerebrospinal fluid (CSF) and amyloid PET analysis is a widely used method to detect these biomarkers, offering high diagnostic accuracy in detecting AD [5]. Nevertheless, its application is limited due to high cost, restricted accessibility, and invasive procedures [6]. To address these limitations, blood-based biomarkers have gained attention for AD screening and monitoring and offer advantages in terms of easy accessibility, affordable cost, and patient comfort [7].

Furthermore, among various Aβ species, oligomeric Aβ is considered the most neurotoxic form, closely linked to AD pathology and synaptic dysfunction. As the disease progresses, amyloid-beta oligomers (AβOs) further lead to cellular degeneration and neuronal death, which are associated with dementia [8]. Due to their earlier pathological impact and higher neurotoxicity, AβOs hold greater potential as an early screening biomarker for AD compared to Aβ_1-42_ and Aβ_1-40_. As such, they may represent a key target for the development of AD biomarkers [9].

Although AβO is considered the most neurotoxic form of Aβ plaques in early AD stage, most existing biomarkers for detecting AD have focused on total Aβ_1–42_, Aβ_1–40_ levels [10] and the Aβ_1-42_/Aβ_1-40_ ratio [11]. Nevertheless, most research on plasma biomarkers for AD has been conducted in Western populations. There is a critical lack of biomarker studies in East Asian populations [12]. These data have shaped current reference and diagnostic values. In particular, data from Taiwanese individuals are scarce, limiting the generalizability of current findings. Therefore, studies involving East Asian populations are necessary to develop biomarker profiles that reflect region-specific genetic and environmental factors. This study aims to explore the association between plasma AβOs and their potential utility as early biomarkers to screen AD.

## 2. Results

Table 1 presents the demographic and cognitive characteristics of the participants. A total of 32 individuals were enrolled, including 16 patients with Alzheimer’s disease (AD) and 16 normal controls (NCs). All participants in both groups were female. The APOE ε4 allele was present in 50% of the AD group and 25% of the NC group, but the difference was not statistically significant (*p* = 1.0). There were no significant differences in the mean ages, which were 80.8 ± 4.3 years in the AD group and 76.5 ± 6.3 years in the NC group (*p* = 0.051). The average years of education were significant lower in the AD group (3.9 ± 4.5) than in the NC group (8.1 ± 4.5), (*p* = 0.02). The MMSE score in the AD group was significantly lower (13.5 ± 8.0) compared to the NC group (23.8 ± 4.6,), (*p* = 0.001).

**Figure 1:**
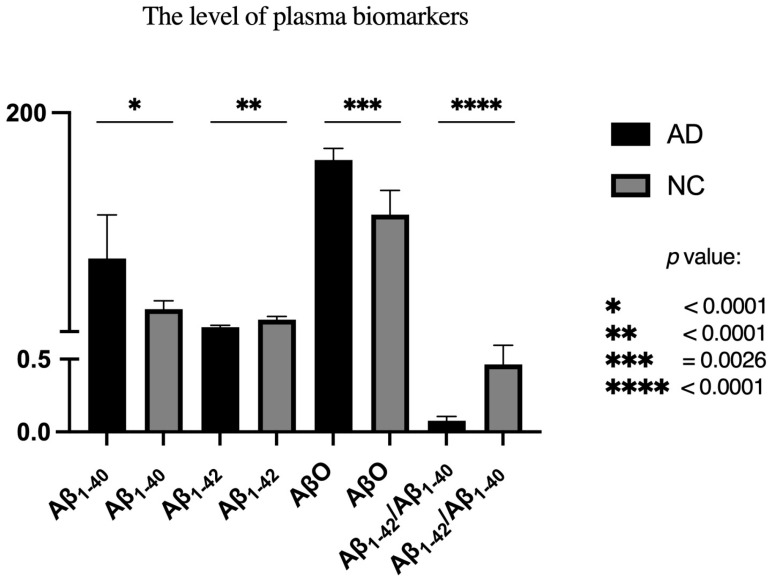
Comparison of plasma biomarkers in both groups.

Our data have also revealed significant differences in plasma Aβ_1-40_ (*p* < 0.001), Aβ_1-42_ (*p* < 0.001), and AβO (*p* = 0.003) concentrations between the AD and NC groups. Specifically, AD patients exhibited elevated Aβ_1-40_ and AβO levels, while Aβ_1-42_ was significantly lower compared to NC. As a result, the NC group showed a higher Aβ_1-42_/Aβ_1-40_ ratio. (Table 2) (Figure 1).

We also observed a strong positive correlation between AβO and Aβ_1-40_ (*p* = 0.003), and a negative correlation with the Aβ_1-42_/Aβ_1-40_ ratio (*p* = 0.002). In contrast, no significant correlation was found between AβO and Aβ_1-42_ alone (*p* = 0.766) (Table 3).

Furthermore, correlations between AβO and cognitive measures, including MMSE and CDR^®^-SB, were not statistically significant in the MMSE test (*p* = 0.2252) and in CDR^®^-SB (*p* = 0.0633); the results may be attributed to the relatively small sample size and limited statistical power of the present study.

## 3. Discussion

This study showed plasma AβO could be a potential biomarker for early screening in AD, owing to significant alterations in plasma Aβ biomarkers between AD patients and NCs (*p* = 0.003). Specifically, levels of Aβ_1-40_ and AβO were significantly elevated, while Aβ_1-42_ and the Aβ_1-42_/Aβ_1-40_ ratio were reduced (*p* < 0.05). Correlation analysis further demonstrated a significantly positive correlation between AβO and Aβ_1-40_ levels and a negative association between AβO and the Aβ_1-42_/Aβ_1-40_ ratio. These findings imply a potential mechanistic link between increased Aβ production or reduced clearance and AβO formation.

Another finding is that higher plasma AβO levels might be associated with poorer cognitive performance. This observation is consistent with the notion that soluble AβOs exert neurotoxic effects that disrupt synaptic function and contribute to cognitive decline in Alzheimer’s disease. Although the current findings did not reach significant difference for several reasons, the direction of the associations highlights the potential of AβO as a biomarker.

The elevated plasma AβO levels in AD patients may reflect early synaptic dysfunction, given the neurotoxic and cognitively deleterious effects of AβO. The decreased Aβ_1-42_/Aβ_1-40_ ratio, consistent with prior studies, is associated with greater cerebral Aβ_1-42_ deposition. Collectively, these findings support the utility of plasma AβO as an early screening biomarker for AD, complementing traditional measurements of Aβ_1-42_ and Aβ_1-40_. Longitudinal studies are needed to explore the temporal dynamics and predictive value of these biomarkers in AD progression.

In agreement with prior studies, our study found consistent alterations in plasma Aβ profiles among individuals with AD. Several recent studies across different populations have reported increased levels of Aβ_1-40_ [13] and AβO [14]. These changes likely reflect early amyloid dysregulation, occurring prior to the manifestation of clinical symptoms. The reproducibility of elevated Aβ_1-40_ and AβO levels across cohorts further supports their value as practical and reliable blood-based biomarkers for early AD detection and differentiation. Additionally, the findings of this study align with previous research using PET and CSF biomarkers, which consistently report increased Aβ_1-40_ and decreased Aβ_1-42_ levels in AD patients [15,16]. The observed elevation in plasma AβO levels is also consistent with prior evidence, suggesting that AβO contributes to synaptic dysfunction and cognitive decline earlier than fibrillar Aβ deposition [17]. The high solubility of AβO [18] and its interactions with synaptic receptors [19] suggest it may serve as a sensitive biomarker in the preclinical stages of AD, even before Aβ_1-42_ and Aβ_1-40_ exhibit disease-causing changes.

Furthermore, Spearman’s correlation in our study analysis demonstrated a positive correlation between AβO and Aβ_1-40_ levels, and a negative association between AβO and the Aβ_1-42_/Aβ_1-40_ ratio. This inverse correlation may reflect the independent decline of Aβ_1-42_, potentially due to its higher propensity for aggregation and lower solubility, which differentiates its behavior from that of AβO [20].

The absence of correlation between AβO and Aβ_1-42_ is due to the stronger tendency of Aβ_1-42_ to aggregate and deposit in the brain [21], which also increases its neurotoxic potential. As Aβ_1-42_ accumulates intracerebrally, its plasma level is reduced. Consequently, plasma AβO is likely derived mainly from the aggregation of plasma Aβ_1-40_, thereby showing a positive correlation with Aβ_1-40_ but not with Aβ_1-42_. In addition, Aβ_1-42_ accounts for only approximately 5% of total Aβ in the brain [22]. This relatively small baseline proportion further limits the amount of Aβ_1-42_ that can reach the plasma once deposition occurs. As a result, patients with early Alzheimer’s disease exhibit reduced plasma Aβ_1-42_ levels, along with a corresponding decline in the Aβ_1-42_/Aβ_1-40_ ratio.

A key strength of this study lies in its multi-biomarker approach, incorporating Aβ_1-40_, Aβ_1-42_, AβO, and the Aβ_1-42_/Aβ_1-40_ ratio to provide a comprehensive assessment of plasma Aβ dynamics in AD. Additionally, this is among the first studies to evaluate plasma AβO levels in a Taiwanese population, offering insights into potential ethnic differences in AD biomarker expression.

However, the study has several limitations. First, the small sample size constrains the statistical power of the findings. Specifically, the small sample size increases the risk of spurious correlation driven by random sampling variation or the influence of outliers. This phenomenon implies that the statistical difference may be overestimated compared to the true population parameter. This study also has limitations regarding the control of factors, such as education, comorbidities, and medication history, which were not included in the current analysis. Our primary objective at this stage was to establish the initial association. Therefore, future research with larger, independent cohorts is essential to incorporate these clinical variables to confirm the validity of this correlation and refine our findings.

Second, the all-female participants limit the generalizability of our findings to male populations. Emerging evidence suggested that biological sex influences both AD risk and pathology. A female population exhibits a higher Aβ accumulation rate compared to a male one [23]. Therefore, the AβO dynamics showed in our findings may be specific to the female population, and we cannot infer similar results to the whole AD population without further verification. Future studies should aim for a sex-balanced design to investigate whether the AβO mechanisms observed in this study are consistent across biological sexes.

Third, the absence of CSF and PET imaging comparisons prevents direct confirmation of AD pathology. Given that CSF Aβ_1-42_ changes typically precede PET-detectable amyloid deposition, future studies should investigate whether plasma Aβ biomarkers reflect similarly early pathological changes. Lastly, larger, more diverse cohorts and the inclusion of imaging and CSF data are essential to validate the diagnostic utility of plasma Aβ biomarkers and understand their role in AD progression.

Finally, the validation of the method itself detecting AβO through ELISA should be examined more seriously and be reported together with the pathological correlation to AD in a large-scale clinical study, although several small-scale studies have been conducted in the clinical trial registration stage.

## 4. Materials and Methods

### 4.1. Subjects

This study is cross-sectional and utilizes data from the Dementia Cohort at Kaohsiung Municipal Ta-Tung Hospital (KMTTH). All procedures for recruiting participants were carried out in accordance with the Helsinki Declaration and were approved by the Institution Review Board of Kaohsiung Medical University Hospital (KMUH-IRB-990301, KMUHIRB-SV(I)-20190025, KMUHIRB-SV(I)-20210067). Sixteen patients diagnosed clinically with dementia due to AD by NINCDS-ADRDA criteria [24] and cognitively normal controls (NCs) were recruited. Demographic information, including biological sex, age, and years of education, as well as results from neuropsychological assessments, were collected for all participants. In addition, plasma concentrations of AβO, Aβ_1-40_, and Aβ_1-42_ were measured. These participants were confirmed by an experienced physician to have a Clinical Dementia Rating^®^ (CDR^®^) score of zero [25] and an AD8 score below 2 [26] and had no family history of Alzheimer’s disease (AD). The NC individuals were volunteers chosen from outpatients at a neurology clinic. None of the enrolled subjects carried any pathological mutations linked to AD or familial AD. Both participants and their family members were briefed on the study details. All participants, or their legal representatives, provided written informed consent before entering this study. Patient and NC data regarding biological sex, age, education years, and neuropsychological tests were recorded. Additionally, plasma concentrations of amyloid-beta peptides were measured.

### 4.2. Neuropsychological Tests

A comprehensive set of neuropsychological assessments was administered to all participants, including the Mini-Mental State Examination (MMSE) [5,27], as well as the Clinical Dementia Rating^®^–Sum of Boxes (CDR^®^-SB) [28]. These measures were used to evaluate clinical status, depressive symptoms, and cognitive functioning. All assessments were conducted by a senior neuropsychologist in conjunction with an experienced physician, with additional information obtained from a knowledgeable collateral informant.

### 4.3. DNA Preparation

Genomic DNA was isolated from 5 mL of peripheral whole blood collected in EDTA-containing tubes to prevent coagulation. Extraction was carried out using the PureLink™ Genomic DNA Mini Kit (Invitrogen, Waltham, MA, USA; K1820-02) in accordance with the manufacturer’s protocol. The resulting DNA samples were stored at −20 °C until subsequent analyses.

### 4.4. Apolipoprotein E Genotyping

Apolipoprotein E (APOE) genotyping was carried out in all participants using a real-time PCR assay based on the TaqMan platform (Applied Biosystems^®^ by Life Technologies, N8010560). The gene copy number for APOE was assessed with commercially available TaqMan Copy Number Assays specific to Apolipoprotein C-I (Assay IDs: C_3084793_20 and C_904973_10). For each TaqMan single-nucleotide polymorphism (SNP) genotyping reaction, the mixture consisted of 3 μL of TaqMan Genotyping Master Mix (Applied Biosystems™, Waltham, MA, USA; P/N 4371355) and 1 μL of genomic DNA (10 ng/μL). PCR amplification was performed using the Applied Biosystems™ 7500 Real-Time PCR System under the following thermal profile: 50 °C for 2 min, 95 °C for 10 min, and 40 cycles of 95 °C for 15 s and 60 °C for 1 min.

### 4.5. Blood Sampling

To compare plasma biomarkers such as AβO, Aβ_1-40_, and Aβ_1-42_ between the AD and NC groups, antibodies for oligomer detection were purchased from Shanghai Jinze Biotechnology Co., Ltd. (China) for oligomers analysis, and other ELISA kits (Human Amyloid β (1–40) Assay Kit–Invitrogen, code number KHB3481, Human Amyloid β (1–42) Assay Kit–Invitrogen, code number, KHB3441) were applied. A previous clinical trial, with the clinical trial number ChiCTR2100046054 and IRB number ChiCTR2100046054, has shown the clinical efficacy of using this ELISA oligomer kit in screening and monitoring in Alzheimer’s disease. That clinical trial recruited 70 patients with mild to moderate AD who were diagnosed and treated with donepezil (10 mg/day) for over 12 months. Baseline demographic data, hippocampal MRI imaging, serum biomarker levels of amyloid beta oligomer (AβO) and P-Tau181, and Mini-Mental State Examination (MMSE) scores were analyzed. Its results have shown that after 12 months of treatment follow-up, the MMSE scores in the entire cohort significantly decreased from baseline (17.0 ± 7.5) to (14.3 ± 8.5) (*p* < 0.001). Higher baseline serum AβO levels, regarded as AβO > 110 pg/m, were associated with faster cognitive decline during subsequent treatment, with a 4.72 score decline in MMSE within 12 months. In contrast, the lower AβO levels, regarded as AβO ≤ 110 pg/m, were associated with less cognitive decline, with a 1.45 score decline in MMSE within 12 months. The original data is being prepared for submission.

Aβ monomers contain a specific and unique epitope, whereas oligomers harbor multiple copies of this epitope. This method applied in the study has employed a patented antibody (Patent No. ZL202510139096.8) targeting the aforementioned unique epitope for both capture and detection. One portion of the antibody is immobilized on a microplate to serve as the capture antibody, while another portion is conjugated with horseradish peroxidase (HRP) to function as the detection antibody. Monomeric proteins, which possess only a single epitope, can be recognized by the capture antibody immobilized on the plate surface. Upon the addition of the detection antibody–HRP conjugate, since the single epitope is already occupied, monomeric proteins cannot be detected. In contrast, oligomers with multiple epitopes can simultaneously bind both the capture and detection antibodies, thereby generating a detectable signal [29,30].

### 4.6. Statistical Analysis

Data are presented as mean ± standard deviation (SD) for quantitative variables and as n (%) for qualitative variables. Comparisons between the AD and NC groups were conducted using the chi-squared test for categorical variables (e.g., sex and APOE ε4 carrier status) and the Mann–Whitney U test for continuous variables. Statistical significance was defined as *p* < 0.05, with results reported as either greater than (>) or less than (<) this threshold where applicable. Spearman’s rank correlation analysis was performed to evaluate the correlation matrix.

## 5. Conclusions

This study demonstrates that elevated plasma AβO levels, alongside increased Aβ_1-40_ and a decreased Aβ_1-42_ and Aβ_1-42_/Aβ_1-40_ ratio, are significantly associated with AD. These alterations suggest that plasma AβO is a promising biomarker for early detection of AD and may provide diagnostic value beyond traditional Aβ measurements. The observed correlations between Aβ_1-40_, AβO, and the Aβ_1-42_/Aβ_1-40_ ratio further underscore a potential mechanistic pathway involving increased Aβ production and oligomerization. Notably, the lack of strong correlation between Aβ_1-42_ and AβO suggests that Aβ_1-42_ dynamics may involve distinct, yet unidentified, biological processes. While these results support the potential utility of plasma AβO in AD screening, the cross-sectional nature of this study limits causal inference. Longitudinal studies with larger, heterogeneous populations are warranted to clarify the temporal dynamics and predictive accuracy of plasma Aβ biomarkers in AD.

## Figures and Tables

**Table 1:** Demographic and clinical characteristics of recruited participants.

Characteristics	AD (*n* = 16)	NC (*n* = 16)	*p* Value
Gender, female (%)	16 (100)	16 (100)	1.0
APOE ε4-positive (%)	8 (50)	4 (25)	0.144
Age (years) mean ± SD	80.8 ± 4.3	76.5 ± 6.3	0.051
Education (years) mean ± SD	3.9 ± 4.5	8.1 ± 4.5	0.020
MMSE CDR^®^	13.5 ± 8.0	23.8 ± 4.6	0.001
CDR^®^ 0.5 (%) CDR^®^ 1 (%) CDR^®^ 2 (%)	2 (12.5%) 12 (75%) 2 (12.5%)		

Abbreviations: AD, Alzheimer’s disease; NC, normal control; APOE, apolipoprotein E; MMSE, Mini-Mental State Examination; CDR^®^, Clinical Dementia Rating^®^.

**Table 2:** The level of plasma biomarkers in recruited participants.

	AD (*n* = 16)	NC (*n* = 16)	*p* Value
Aβ_1-40_ (pg/mL)	67.4 (44.2–107.2)	21.4 (19.6–29.1)	<0.001
Aβ_1-42_ (pg/mL)	5.0 (3.0–6.7)	11.9 (8.4–15.0)	<0.001
AβO(pg/mL)	157.1 (133.2–167.5)	107.3 (81.6–129.5)	0.003
Aβ_1-42_/Aβ_1-40_	0.08 (0.05–0.1)	0.5 (0.4–0.7)	<0.001

Abbreviations: AD, Alzheimer’s disease; NC, normal control; Aβ, amyloid beta; AβO, amyloid-beta oligomer.

**Table 3:** Spearman’s correlation among AβO with Aβ_1-40_, Aβ_1-42_, and Aβ_1-42_/Aβ_1-40_.

Traditional Aβ Proteins	AβO (*n* = 16)	
	Spearman’s Correlation Coefficient (r)	*p* Value
Aβ_1-40_ (pg/mL)	0.697	0.003 **
Aβ_1-42_ (pg/mL)	−0.081	0.766
Aβ_1-42_/Aβ_1-40_	−0.706	0.002 **

AβO and plasma proteins in all groups were assessed using the Spearman’s correlation test. ** *p* < 0.05, statistically significant.

## Data Availability

Data supporting the findings of this study are available from the corresponding author upon reasonable request but are not publicly accessible due to privacy and ethical restrictions.

## Abbreviations

The following abbreviations are used in this manuscript:

CDR^®^-SB	Clinical Dementia Rating^®^-Sum of Boxes
NIA-AA	the National Institute on Aging-Alzheimer’s Association
MMSE	Mini-Mental State Examination
APOE	Apolipoprotein E
NC	Normal Control
AD	Alzheimer’s disease
Aβ	Amyloid-Beta
